# Immunome database for marsupials and monotremes

**DOI:** 10.1186/1471-2172-12-48

**Published:** 2011-08-19

**Authors:** Emily SW Wong, Anthony T Papenfuss, Katherine Belov

**Affiliations:** 1 Faculty of Veterinary Sciences, University of Sydney, NSW 2006, Australia; 2 Bioinformatics Division, The Walter and Eliza Hall Institute for Medical Research, Parkville, Victoria 3050, Australia

## Abstract

**Background:**

To understand the evolutionary origins of our own immune system, we need to characterise the immune system of our distant relatives, the marsupials and monotremes. The recent sequencing of the genomes of two marsupials (opossum and tammar wallaby) and a monotreme (platypus) provides an opportunity to characterise the immune gene repertoires of these model organisms. This was required as many genes involved in immunity evolve rapidly and fail to be detected by automated gene annotation pipelines.

**Description:**

We have developed a database of immune genes from the tammar wallaby, red-necked wallaby, northern brown bandicoot, brush-tail possum, opossum, echidna and platypus. The resource contains 2,235 newly identified sequences and 3,197 sequences which had been described previously. This comprehensive dataset was built from a variety of sources, including EST projects and expert-curated gene predictions generated through a variety of methods including chained-BLAST and sensitive HMMER searches. To facilitate systems-based research we have grouped sequences based on broad Gene Ontology categories as well as by specific functional immune groups. Sequences can be extracted by keyword, gene name, protein domain and organism name. Users can also search the database using BLAST.

**Conclusion:**

The Immunome Database for Marsupials and Monotremes (IDMM) is a comprehensive database of all known marsupial and monotreme immune genes. It provides a single point of reference for genomic and transcriptomic datasets. Data from other marsupial and monotreme species will be added to the database as it become available. This resource will be utilized by marsupial and monotreme immunologists as well as researchers interested in the evolution of mammalian immunity.

## Background

Recently, two marsupial genomes and one monotreme genome have been sequenced: the grey short-tailed opossum (*Monodelphis domestica*; 7× coverage) [1], the tammar wallaby (*Macropus eugenii*; 2×) (in prep.), and the platypus (*Ornithorhynchus anatinus*; 6×) [2]. Marsupial and monotreme lineages branched off approximately 148 My and 166 My ago from the lineage leading to eutherian mammals [3]. They hold a unique evolutionarily position providing a link to the reptilian phase of our ancestry. Combined with their unusual biological traits, they are capable of providing important insights to our understanding of mammalian biology and evolution.

Genome sequencing has generated huge amounts of genomic data. This has expedited the identification of genes in these species. Despite the availability of genome assemblies, only the most phylogenetically conserved immune genes have been identified using automated gene annotation pipelines. Genes involved in the immune response are subject to intense selective pressure due to the need to overcome pathogenic challenges. As a result, it is common for immune genes, particularly those with immunomodulatory roles, to show very low levels of sequence conservation between species [4,5]. This has lead to many key immune molecules being missed by the Ensembl [6] and NCBI's Gnomon http://www.ncbi.nlm.nih.gov genome annotation platforms. Less than a third of all opossum immune genes that were annotated using specialized search strategies by Wong et al. 2006 [7], Belov et al. 2006 [8] and Belov et al. 2007 [9], were predicted by the Ensembl pipeline [6]. Aside from high levels of sequence divergence, many immune gene families have also evolved through rapid successions of gene loss and gain, resulting in a lack of direct orthologs. Hence, these genes are difficult to characterize through local pairwise similarity search algorithms, such as BLAST [10], which use a single gene sequence to query a database.

To overcome the lack of annotated sequence information for immune genes, targeted, manually-curated strategies were applied [7,9,11,12]. Identification of the most highly divergent sequences required an intensive combination of strategies incorporating hidden Markov model searches, exploitation of conserved syntenic regions, sensitive local search algorithms and gene prediction integrating extrinsic information [7,9,11,12]. Less divergent genes missed by Ensembl could be identified and annotated using chained-BLAST searches [9].

Here, we present a database of curated marsupial and monotreme immune sequences. We have included novel predicted and expressed sequences as well as previously annotated genes [7,9,11-45]. Examples of gene groups represented in the database include chemokines, interleukins, Natural Killer (NK) receptors, Major Histocompatibility Complex (MHC) antigens, surface receptors, antimicrobial peptides. Annotations derived from a transcriptomic analysis on a primary lymphoid organ have also been included [46]. Many of these genes (e.g. 209 expressed tammar genes) have not been annotated by Ensembl and their sequences are not curated by other public databases. The database consists of a simple interface, and features several methods for users to query the sequences. On entry to the database, sequences were further annotated to provide searchable functional information. Availability of a comprehensive gene set assists large-scale projects such as transcriptomic analysis and microarray studies. Also, it facilitates the development of marsupial- and monotreme-specific reagents allowing for detailed analyses of metatherian and prototherian immune responses.

## Construction and content

IDMM was implemented using the Python web framework Django (version 1.1) [47] with a SQLite3 (version 3.6.3) database [48]. Data can be easily updated by approved managers through a simple web interface. Once sequences are added, they are automatically matched to HGNC names and GO terms through a BLAST search. Amino acid sequences are additionally searched against the Conserved Domain Database (CDD) [49] to create protein domain annotations. Sequences are stored in FASTA format and are identified by their sequence header description which includes the gene name and species name.

### Database content and data source

A total of 2,935 genes, 602 expressed (538 tammar wallaby, 24 opossum, 16 platypus, 11 echidna, 6 red-necked wallaby, 4 brushtail possum and 3 bandicoot) and 2,333 predicted (1,639 opossum, 694 platypus), are currently stored in the database. The database includes 1,985 published sequences. We have integrated data from various published resources, which include expressed and predicted genes from opossum (1,663) [7-9,18,34,35,38,39,43-45,50], tammar (37) [21-26,33,35-37,45], brushtail possum (4) [18,20,27-29,40] echidna (11) [14,17,19,30,32], bandicoot (3) [15], red-necked wallaby (6) [42] and platypus (261) [11-14,16,18,19,31,41]. Manually annotated gene families include: major histocompatibility complex (MHC), leucocyte receptor complex (LRC), cytokine, defensin, cathelicidin, natural killer complex (NKC) and Fc receptor genes. Both opossum and platypus sequences were annotated using a curated list of human immune genes from the IRIS database [51]. For predicted genes, candidate gene regions were first identified using either BLAST [10] or HMMER hidden Markov model [52] searches. Following this, best hits were either concatenated into genes or used to predict a full gene model using a gene prediction program. 516 wallaby genes were annotated based on opossum genes identified in Wong et al. 2006 [7] and Belov et al. 2007 [9]. Of these, at least 217 were not annotated by Ensembl (version 58). Wallaby reads were derived from the pyrosequencing of wallaby thymus transcriptomes and annotated using the wallaby (v1.0) genome assembly [46]. For each annotated wallaby gene there were often multiple, overlapping reads; these were assembled and included in the database (1,786 wallaby reads in total). 449 platypus gene sequences were obtained by concatenation of the highest-scoring IRIS BLAST hits against the platypus genome assembly (v5.0) (Unpublished). Of these, 366 genes were not annotated by Ensembl (version 58).

### Sequence annotation

All reads were defined by their species name, a gene symbol, the method of identification and sequence type (nucleotide or amino acid). To facilitate the retrieval of genes associated with specific immune roles, we categorized genes based on nine functional terms. These include the broad categories of humoral and cellular immunity and components of the innate (inflammation and complement system) and adaptive (antigen processing and presenting and phagocytosis) immune responses, as well as genes with regulatory functions such as chemokines and transcription factors. To provide additional sequence-based and functional information, all sequences were automatically annotated upon submission to the database. Automatic annotation was performed by searching the human SWISS-PROT [53] database at NCBI [54] with the submitted sequences using the network BLAST client (netblast) [10]. This resulted in the association of sequences with the official human gene names [55], GO ontology terms [56], and, for protein sequences, domain names. The accession of the best hit from each BLAST search was retrieved and matched to a list of pre-generated accession-specific tags if the E-value was less than 1e-3. These tags were linked to human gene names and gene ontology annotations using Entrez Gene data [57].

## Utility and Discussion

### User interface

**Figure 1:**
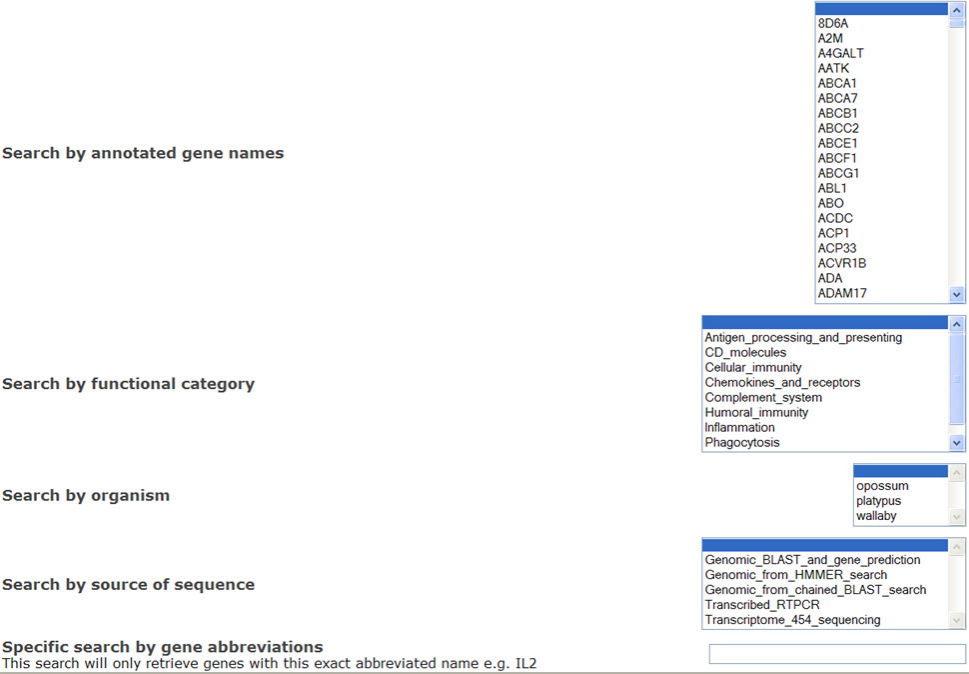
**Main search page of IDMM**.

**Figure 2:**
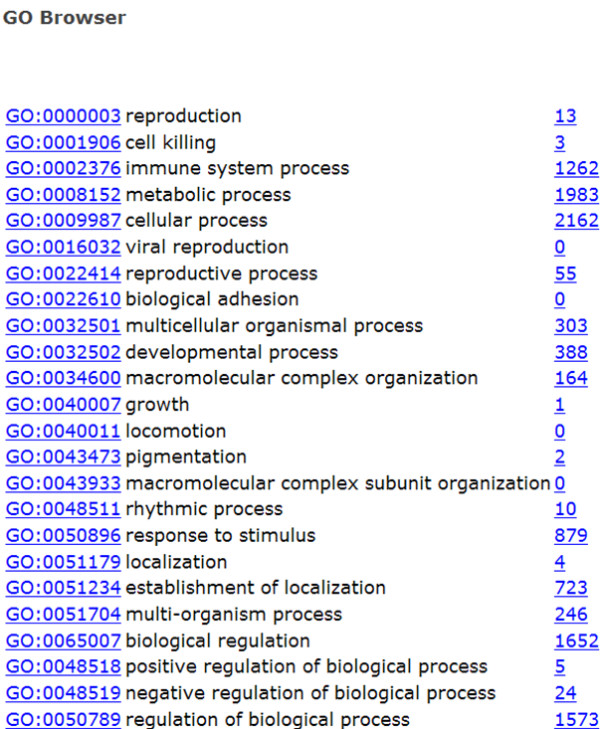
**Gene Ontology (GO) tree browser**.

Users can interrogate the database and retrieve gene sequences through a variety of simple query tools. The search interface spans three webpages. From the main page, users can query the database through keyword, organism name, human gene name, protein domain name and by the method through which sequences were obtained (Figure 1). A link exists to a GO term browser where terms can be examined in a tree structure that supports the natural relationships between GO terms (Figure 2). Finally, the BLAST program is implemented for users to search against sequences in the database.

### Search by curated gene symbols

All gene names determined through annotation can be browsed. All sequences have been annotated with a gene name based on the human gene symbol, with the exception of lineage-specific expansions, such as NKC genes and MHC genes. Characterized species-specific expansions (i.e. without human one-to-one orthologs) are labelled using the gene family name followed by a unique set of numeric identifiers.

### Keyword search

A simple keyword search permits users to query the database using any string of characters from any description line in FASTA sequences, human gene descriptions and GO names. All FASTA descriptions contain the common name of the species from which the sequences were derived. In addition to terms present in the FASTA header description, users may also search terms generated by automatic annotation which include full gene name (in addition to HGNC symbol) and GO terms. Only sequences of high similarity (E-value < 1e-3) to human genes were automatically annotated. Two keyword searches are available: one for exact but case-insensitive match in sequence headers only and one which matches all terms containing the keyword from all associations, including, for example, GO descriptions.

### Search by sequence identification method

Sequence retrieval via the initial sequence identification method (e.g. BLAST) allows simple discrimination between expressed and predicted genes. It is important to note that while chained high scoring BLAST alignments may provide more sequence information, the predicted sequence may not be identical to the actual transcribed sequence. We have also provided information on the identification method used on each sequence label.

### Search by HGNC gene symbols

To facilitate the retrieval of marsupial and monotreme homologs to human genes, a list of human gene symbols is available for browsing. We queried marsupial and monotreme database sequences against all human proteins and linked the best hits based on the E-value. The resultant annotations are, in effect, reciprocal best hits of predicted genes. By comparison of gene symbols, users can rapidly determine the accuracy of an ortholog assignment. This strategy provides a measure of the level of confidence in the assigned gene name.

### Search by conserved protein domains

To facilitate rapid identification of gene family members, users can search for sequences based on annotated protein functional units from the Conserved Domain Database (CDD). CDD names can be browsed by list and by hyperlinks via 'tag cloud'. Conserved domain annotations are only available for amino acid sequences.

### Search based on GO terms

Users may interrogate biological and molecular functional processes and structural components through a GO browser (Figure 2). The browser follows the tree-like hierarchy of GO data by linking general terms to specific terms. The GO terms associated with monotreme and marsupial sequences are inferred through sequence similarity to human Entrez gene annotations. For each term the number of associated database sequences is located after the GO name. By clicking on this number users can extract all associated sequences. Note that Entrez GO terms often miss higher level terms which will underestimate the number of genes in a category. Therefore, it is advisable to browse through GO child terms.

### Search through the BLAST interface

Users can direct BLAST queries against the sequence database. Users can perform nucleotide, translated nucleotide and protein searches. Results are presented in standard BLAST text output format.

### Sequence retrieval

With the exception of BLAST searches, sequences are viewed through a standard retrieval interface. FASTA headers uniquely identify each sequence. In addition to the option of retrieving reads individually, users may choose to retrieve all identified sequences at once. Users can also fetch all associated annotations for each sequence. An option to display amino acid or nucleotide sequences is available.

## Conclusion

Targeted search strategies for immune genes and gene families have led to the annotation of previously unidentified marsupials and monotreme genes in the recent genome assemblies of the opossum, tammar wallaby and platypus. Genes involved in immunity are generally poorly annotated in genome assemblies due to their high rate of sequence divergence and gene duplications. This high sequence divergence of marsupial and monotreme immune genes also renders them difficult to isolate with classical lab techniques. IDMM provides easy access to marsupial and monotreme immune sequences. It hosts a catalogue of novel and integrated sets of published genes, searchable through a simple-to-use and fast interface. The availably of these sequences will facilitate the development of species-specific immunological reagents, enabling accurate studies of immune responses in these species. This database will be useful for comparative studies of immunity.

## Availability and requirements

IDMM is publicly available at http://hp580.angis.org.au/tagbase/gutentag/.

## Authors' contributions

EW sourced and identified the sequences, designed and implemented the database and web interface. AP, KB and EW conceived the concept. EW wrote the manuscript and KB and AP edited the manuscript. All authors read and approved the final manuscript.

## References

[B1] Mikkelsen TS Wakefield MJ Aken B Amemiya CT Chang JL Duke S Garber M Gentles AJ Goodstadt L Heger A Genome of the marsupial Monodelphis domestica reveals innovation in non-coding sequences Nature 2007 447 7141 167 177 10.1038/nature05805 17495919

[B2] Warren WC Hillier LW Marshall Graves JA Birney E Ponting CP Grutzner F Belov K Miller W Clarke L Chinwalla AT Genome analysis of the platypus reveals unique signatures of evolution Nature 2008 453 7192 175 183 10.1038/nature06936 18464734 PMC2803040

[B3] Bininda-Emonds OR Cardillo M Jones KE MacPhee RD Beck RM Grenyer R Price SA Vos RA Gittleman JL Purvis A The delayed rise of present-day mammals Nature 2007 446 7135 507 512 10.1038/nature05634 17392779

[B4] Zelus D Robinson-Rechavi M Delacre M Auriault C Laudet V Fast evolution of interleukin-2 in mammals and positive selection in ruminants Journal of Molecular Evolution 2000 51 3 234 244 11029068 10.1007/s002390010085

[B5] Kaiser P Poh TY Rothwell L Avery S Balu S Pathania US Hughes S Goodchild M Morrell S Watson M A genomic analysis of chicken cytokines and chemokines Journal of Interferon & Cytokine Research: The Official Journal of the International Society for Interferon and Cytokine Research 2005 25 8 467 484 16108730 10.1089/jir.2005.25.467

[B6] Curwen V Eyras E Andrews TD Clarke L Mongin E Searle SMJ Clamp M The Ensembl automatic gene annotation system Genome Research 2004 14 5 942 950 10.1101/gr.1858004 15123590 PMC479124

[B7] Wong E Young L Papenfuss A Belov K In silico identification of opossum cytokine genes suggests the complexity of the marsupial immune system rivals that of eutherian mammals Immunome Research 2006 2 1 4 4 10.1186/1745-7580-2-4 17094811 PMC1660534

[B8] Belov K Deakin JE Papenfuss AT Baker ML Melman SD Siddle HV Gouin N Goode DL Sargeant TJ Robinson MD Reconstructing an Ancestral Mammalian Immune Supercomplex from a Marsupial Major Histocompatibility Complex PLoS Biology 2006 4 3 e46 EP--e46 EP -10.1371/journal.pbio.0040046 PMC1351924 16435885

[B9] Belov K Sanderson CE Deakin JE Wong ES Assange D McColl KA Gout A de Bono B Barrow AD Speed TP Characterization of the opossum immune genome provides insights into the evolution of the mammalian immune system Genome Res 2007 17 7 982 991 10.1101/gr.6121807 17495011 PMC1899125

[B10] Altschul SF Madden TL Schaffer AA Zhang J Zhang Z Miller W Lipman DJ Gapped BLAST and PSI-BLAST: a new generation of protein database search programs Nucleic Acids Res 1997 25 17 3389 3402 10.1093/nar/25.17.3389 9254694 PMC146917

[B11] Wong ESW Papenfuss AT Miller RD Belov K Hatching time for monotreme immunology Australian Journal of Zoology 2009 57 4 185 198 10.1071/ZO09042

[B12] Wong ES Sanderson CE Deakin JE Whittington CM Papenfuss AT Belov K Identification of natural killer cell receptor clusters in the platypus genome reveals an expansion of C-type lectin genes Immunogenetics 2009 61 8 565 579 10.1007/s00251-009-0386-7 19597809

[B13] Whittington CM Papenfuss AT Bansal P Torres AM Wong ES Deakin JE Graves T Alsop A Schatzkamer K Kremitzki C Defensins and the convergent evolution of platypus and reptile venom genes Genome Res 2008 18 6 986 994 10.1101/gr.7149808 18463304 PMC2413166

[B14] Belov K Lam MKP Hellman L Colgan DJ Evolution of the major histocompatibility complex: Isolation of class II beta cDNAs from two monotremes, the platypus and the short-beaked echidna Immunogenetics 2003 55 6 402 411 10.1007/s00251-003-0598-1 12942212

[B15] Baker ML Miller RD Evolution of mammalian CD1: marsupial CD1 is not orthologous to the eutherian isoforms and is a pseudogene in the opossum Monodelphis domestica Immunology 2007 121 1 113 121 10.1111/j.1365-2567.2007.02545.x 17244156 PMC2265927

[B16] Johansson J Salazar JN Aveskogh M Munday B Miller RD Hellman L High variability in complementarity-determining regions compensates for a low number of V gene families in the lambda light chain locus of the platypus European Journal of Immunology 2005 35 10 3008 3019 10.1002/eji.200425574 16143985

[B17] Nowak MA Parra ZE Hellman L Miller RD The complexity of expressed kappa light chains in egg-laying mammals Immunogenetics 2004 56 8 555 563 10.1007/s00251-004-0720-z 15448942

[B18] Miska KB Hellman L Miller RD Characterization of beta(2)-microglobulin coding sequence from three non-placental mammals: the duckbill platypus, the short-beaked echidna, and the grey short-tailed opossum Developmental and Comparative Immunology 2003 27 3 247 256 10.1016/S0145-305X(02)00095-2 12590975

[B19] Miska KB Harrison GA Hellman L Miller RD The major histocompatibility complex in monotremes: an analysis of the evolution of Mhc class I genes across all three mammalian subclasses Immunogenetics 2002 54 6 381 393 10.1007/s00251-002-0484-2 12242589

[B20] Belov K Harrison GA Miller RD Cooper DW Molecular cloning of four lambda light chain cDNAs from the Australian brushtail possum Trichosurus vulpecula European Journal of Immunogenetics: Official Journal of the British Society for Histocompatibility and Immunogenetics 2002 29 2 95 99 10.1046/j.1365-2370.2002.00286.x 11918633

[B21] Old JM Deane EM Harrison GA Molecular characterisation of the tammar wallaby (Macropus eugenii) CD3 epsilon chain cDNA Molecular Immunology 2001 38 5 359 364 10.1016/S0161-5890(01)00072-4 11684291

[B22] Daly KA Digby MR Lefévre C Nicholas KR Deane EM Williamson P Identification, characterization and expression of cathelicidin in the pouch young of tammar wallaby (Macropus eugenii) Comparative Biochemistry and Physiology Part B, Biochemistry & Molecular Biology 2008 149 3 524 533 10.1016/j.cbpb.2007.12.002 18248751

[B23] Harrison GA McNicol KA Deane EM Interferon alpha/beta genes from a marsupial, Macropus eugenii Developmental and Comparative Immunology 2004 28 9 927 940 10.1016/j.dci.2004.02.002 15183033

[B24] Harrison GA Deane EM cDNA sequence of the lymphotoxin beta chain from a marsupial, Macropus eugenii (Tammar wallaby) Journal of Interferon & Cytokine Research: The Official Journal of the International Society for Interferon and Cytokine Research 1999 19 10 1099 1102 10547148 10.1089/107999099313028

[B25] Harrison GA Broughton MJ Young LJ Cooper DW Deane EM Conservation of 3' untranslated region elements in tammar wallaby (Macropus eugenii) TNF-alpha mRNA Immunogenetics 1999 49 5 464 467 10.1007/s002510050521 10199924

[B26] Harrison GA Deane EM cDNA cloning of lymphotoxin alpha (LT-alpha) from a marsupial, Macropus eugenii DNA Sequence: The Journal of DNA Sequencing and Mapping 2000 10 6 399 403 10.3109/10425170009015608 10826697

[B27] Wedlock DN Goh LP Parlane NA Buddle BM Molecular cloning and physiological effects of brushtail possum interleukin-1beta Veterinary Immunology and Immunopathology 1999 67 4 359 372 10.1016/S0165-2427(99)00004-5 10206203

[B28] Wedlock DN Aldwell FE Buddle BM Molecular cloning and characterization of tumor necrosis factor alpha (TNF-alpha) from the Australian common brushtail possum, Trichosurus vulpecula Immunology and Cell Biology 1996 74 2 151 158 10.1038/icb.1996.20 8724002

[B29] Cui S Selwood L cDNA cloning, characterization, expression and recombinant protein production of leukemia inhibitory factor (LIF) from the marsupial, the brushtail possum (Trichosurus vulpecula) Gene 2000 243 1-2 167 178 10.1016/S0378-1119(99)00513-2 10675625

[B30] Vernersson M Aveskogh M Hellman L Cloning of IgE from the echidna (Tachyglossus aculeatus) and a comparative analysis of epsilon chains from all three extant mammalian lineages Developmental and Comparative Immunology 2004 28 1 61 75 10.1016/S0145-305X(03)00084-3 12962983

[B31] Parra ZE Arnold T Nowak MA Hellman L Miller RD TCR gamma chain diversity in the spleen of the duckbill platypus (Ornithorhynchus anatinus) Developmental and Comparative Immunology 2006 30 8 699 710 10.1016/j.dci.2005.10.002 16303181

[B32] Belov K Miller RD Ilijeski A Hellman L Harrison GA Isolation of monotreme T-cell receptor alpha and beta chains Immunogenetics 2004 56 3 164 169 15133646 10.1007/s00251-004-0679-9

[B33] Harrison GA Taylor CL Miller RD Deane EM Primary structure and variation of the T-cell receptor delta-chain from a marsupial, Macropus eugenii Immunology Letters 2003 88 2 117 125 10.1016/S0165-2478(03)00072-5 12880681

[B34] Parra ZE Baker ML Schwarz RS Deakin JE Lindblad-Toh K Miller RD A unique T cell receptor discovered in marsupials Proceedings of the National Academy of Sciences of the United States of America 2007 104 23 9776 9781 10.1073/pnas.0609106104 17535902 PMC1887558

[B35] Duncan LG Nair SV Deane EM The marsupial CD8 gene locus: molecular cloning and expression analysis of the alpha and beta sequences in the gray short-tailed opossum (Monodelphis domestica) and the tammar wallaby (Macropus eugenii) Veterinary Immunology and Immunopathology 2009 129 1-2 14 27 10.1016/j.vetimm.2008.12.003 19135263

[B36] Siddle HV Deakin JE Baker ML Miller RD Belov K Isolation of major histocompatibility complex Class I genes from the tammar wallaby (Macropus eugenii) Immunogenetics 2006 58 5-6 487 493 10.1007/s00251-006-0107-4 16568263

[B37] Daly KA Digby M Lefèvre C Mailer S Thomson P Nicholas K Williamson P Analysis of the expression of immunoglobulins throughout lactation suggests two periods of immune transfer in the tammar wallaby (Macropus eugenii) Veterinary Immunology and Immunopathology 2007 120 3-4 187 200 10.1016/j.vetimm.2007.07.008 17727962

[B38] Parra ZE Baker ML Lopez AM Trujillo J Volpe JM Miller RD TCR mu recombination and transcription relative to the conventional TCR during postnatal development in opossums Journal of Immunology (Baltimore, Md: 1950) 2009 182 1 154 163 10.4049/jimmunol.182.1.154 PMC2921273 19109146

[B39] Baker ML Melman SD Huntley J Miller RD Evolution of the opossum major histocompatibility complex: evidence for diverse alternative splice patterns and low polymorphism among class I genes Immunology 2009 128 1 Suppl e418 431-e418-431 19191910 10.1111/j.1365-2567.2008.02994.x PMC2753908

[B40] Adamski FM Demmer J Immunological protection of the vulnerable marsupial pouch young: two periods of immune transfer during lactation in Trichosurus vulpecula (brushtail possum) Developmental and Comparative Immunology 2000 24 5 491 502 10.1016/S0145-305X(00)00012-4 10785274

[B41] Vernersson M Belov K Aveskogh M Hellman L Cloning and structural analysis of two highly divergent IgA isotypes, IgA1 and IgA2 from the duck billed platypus, Ornithorhynchus anatinus Molecular Immunology 2010 47 4 785 791 10.1016/j.molimm.2009.10.007 19913303

[B42] Schneider S Vincek V Tichy H Figueroa F Klein J MHC class II genes of a marsupial, the red-necked wallaby (Macropus rufogriseus): identification of new gene families Molecular Biology and Evolution 1991 8 6 753 766 1775063 10.1093/oxfordjournals.molbev.a040688

[B43] Miller RD Grabe H Rosenberg GH V(H) repertoire of a marsupial (Monodelphis domestica) Journal of Immunology (Baltimore, Md: 1950) 1998 160 1 259 265 9551979

[B44] Lucero JE Rosenberg GH Miller RD Marsupial light chains: complexity and conservation of lambda in the opossum Monodelphis domestica Journal of Immunology (Baltimore, Md: 1950) 1998 161 12 6724 6732 9862702

[B45] Duncan LG Nair SV Deane EM Molecular characterisation and expression of CD4 in two distantly related marsupials: the gray short-tailed opossum (Monodelphis domestica) and tammar wallaby (Macropus eugenii) Molecular Immunology 2007 44 15 3641 3652 10.1016/j.molimm.2007.04.013 17521733

[B46] Wong ES Papenfuss AT Heger A Hsu AL Ponting CP Miller R Renfree MB Gibbs RA Belov K Transcriptomic analysis supports similar functional roles for the two thymuses of the tammar wallaby BMC Genomics in press 10.1186/1471-2164-12-420 PMC3173455 21854594

[B47] Django http://www.djangoproject.com/

[B48] SQLite http://sqlite.org/

[B49] Marchler-Bauer A Panchenko AR Shoemaker BA Thiessen PA Geer LY Bryant SH CDD: a database of conserved domain alignments with links to domain three-dimensional structure Nucleic Acids Res 2002 30 1 281 283 10.1093/nar/30.1.281 11752315 PMC99109

[B50] Miska KB Miller RD Marsupial Mhc class I: classical sequences from the opossum, Monodelphis domestica Immunogenetics 1999 50 1-2 89 93 10.1007/s002510050692 10541813

[B51] Kelley J de Bono B Trowsdale J IRIS: a database surveying known human immune system genes Genomics 2005 85 4 503 511 10.1016/j.ygeno.2005.01.009 15780753

[B52] Eddy SR Profile hidden Markov models Bioinformatics 1998 14 9 755 763 10.1093/bioinformatics/14.9.755 9918945

[B53] Boeckmann B Bairoch A Apweiler R Blatter MC Estreicher A Gasteiger E Martin MJ Michoud K O'Donovan C Phan I The SWISS-PROT protein knowledgebase and its supplement TrEMBL in 2003 Nucleic Acids Res 2003 31 1 365 370 10.1093/nar/gkg095 12520024 PMC165542

[B54] National Center for Biotechnology Information (NCBI) http://www.ncbi.nlm.nih.gov/

[B55] HUGO Gene Nomenclature Committee http://www.genenames.org/

[B56] Ashburner M Ball CA Blake JA Botstein D Butler H Cherry JM Davis AP Dolinski K Dwight SS Eppig JT Gene ontology: tool for the unification of biology. The Gene Ontology Consortium Nat Genet 2000 25 1 25 29 10.1038/75556 10802651 PMC3037419

[B57] Maglott D Ostell J Pruitt KD Tatusova T Entrez Gene: gene-centered information at NCBI Nucleic Acids Res 2007 35 Database D26 31 10.1093/nar/gkl993 PMC1761442 17148475

